# Ice-Houses

**Published:** 1866-04

**Authors:** 


### Ice-Houses

Are beginning to be considered an indispensable appendage to a farmer's house, and, indeed, to every man who owns his premises. They are not a necessity, and where there is a good spring, or never-failing well, they can be very readily dispensed with, especially as they do not contribute to the general health of any family, unless the use of ice is wisely controlled. The free use of ice-water tends to the decay of the teeth prematurely, is liable to produce dangerous inflammations of the stomach, and certainly is the immediate cause of dyspeptic diseases in multitudes of cases, where it is freely indulged in at the regular meals of the day. At the same time, as many will prefer building ice-houses, it is proper here to give some directions in reference to the subject.

That ice keeps better ordinarily above ground than below, and that ventilation is necessary in order to its well-keeping, are two indisputable facts. The more compact the mass of ice is, the longer will it keep; hence plans have been devised of letting a stream of water run slowly into the ice-house after it has been filled, so that all the crevices may be filled up; or where a running stream is available, some persons have arranged to let the water in a foot deep during very cold weather; when this has frozen solid, let in a few inches more, until the house is entirely filled; or it can be done with less trouble and attention if during very severe weather the water is conveyed into the ice-house during the night, by or from a running stream, in a very fine spray, freezing as it falls. There should be a double roof; the under part of the rafters should be boarded closely, and between that and the shingles a space of eight or ten inches or more should be filled up with saw-dust, spent tan-bark, or other porous substance. There should be a space between the straw on the surface of the ice and the roof, for purposes of ventilation, to prevent the air from becoming damp and close, with a wooden chimney of eight or ten inches square piercing the roof, or a sliding panel in the door would answer; the ventilation must not be a current of air. If the eaves of the roof extend a foot or two over the sides, a greater protection is afforded against rain and the rays of the sun. The roof of an ice-house should be steep. Great care should be taken against leakages of this, as well as of all other farm buildings. A cement may be applied with a trowel or case-knife to all leaks in roofs or about chimneys, etc., made thus: Take pure white lead and mix it with boiled oil until it is of the thickness of thin paint, add to this common sand until of the thickness of common mortar; there is perhaps nothing better than this. A space twelve feet in the cellar in every direction, will hold enough ice for a large family.

Ice-houses should be located, as a general rule, on the north side of a hill, if built under ground, so that the ice can be approached on a level with the ground on which it is built. On many farms such a location is impracticable, and the only alternative is to build one on the surface. The general construction should be a wooden frame building, with another outside of it, with a space intervening of from fifteen to thirty inches, which should be filled in with coal-cinders, tan-bark, or, which is better than either, pulverized charcoal. It would be better if the inner building were made of solid timbers close together, and about three inches thick; the outer one, or the shell, may be a common frame, neatly weather-boarded, and kept well painted with white lead, so as to repel the heat of the sun. It will add to the convenience of an ice-house if the bottom, or at least a part of it, is arched, so as to form a place for a larder under this arch, or the drainings of the ice should be made to pass through the dairy or "spring-house."

Many farms have small streams of water running through them. In such cases, the locality for an ice-house should be selected with reference to the convenience of damming this stream near it, before Christmas, in such a way that a lake of a hundred feet or more in diameter, and about two feet deep, may be formed, and properly protected from cattle and all nuisances. This body of water would yield enough ice for a large farm, and by its shallowness would be more certain to yield a crop of ice, because a less degree of cold would be required to freeze it solidly than in a deeper stream, or one which was running, even with a sluggish current. One freezing over would yield thirty or forty one-horse loads of this summer luxury. While the lavish use of ice and ice-water can not but be prejudicial to the health of any family, common ice is one of the most valuable of remedial means in case of sickness in various forms.

To a person burning up with internal fevers ice is a comfort beyond expression.

Swallowing ice freely in small lumps is the chief treatment in inflammation of the stomach.

The constant application of ice, pounded fine, and enveloping the head with it by means of a cushion, or other contrivance, is the most reliable remedy for that dangerous malady, inflammation of the brain, which so often sends its victim to the grave in a few days, or to that living death, the mad-house!!

In all inflammations, whether internal or external, ice diminishes rapidly the size of the blood-vessels, and thus relieves the pain they give when thus swollen by their pressing against the nerves which are always in the neighborhood of the arteries of the system.

Diphtheria, and some of the worst of other forms of sore throat, has been arrested in a very short time by pounding a piece of ice in a bag, then laying the head back, take the lumps and swallow them continuously until relieved, allowing them to be detained in the throat as long as possible, there to melt.

In all forms of diarrhea and dysentery, where there is great thirst, the gratification of which by drinking any liquid increases the malady, are promptly controlled, and in many cases perfectly cured, by simply swallowing as large lumps of ice as possible.

Epilepsy itself, one of the most uncontrollable of human maladies, is said to be treated successfully in London by the application of ice to the spinal portion of the system.

A piece of ice laid on the wrist will often arrest profuse and dangerous bleeding of the nose.

In croup, water as cold as ice can make it, if applied freely and persistently to the throat, neck, and upper part of the chest with a sponge or cloth, often affords an almost miraculous relief, especially if followed by drinking copiously of ice-water, wiping the wetted parts perfectly dry, then wrapping the child closely up in dry flannels, allowing it to fall into a delightful and life-giving slumber.

These statements may induce the farmer to be at pains, if he does conclude to build an ice-house, to have it done in the most thorough manner and after the most approved pattern.

